# Characteristics and outcomes for patients with advanced vaginal or vulvar cancer referred to a phase I clinical trials program: the MD Anderson cancer center experience

**DOI:** 10.1186/s40661-015-0018-x

**Published:** 2015-11-14

**Authors:** Siqing Fu, Naiyi Shi, Jennifer Wheler, Aung Naing, Filip Janku, Sarina Piha-Paul, Jing Gong, David Hong, Apostolia Tsimberidou, Ralph Zinner, Vivek Subbiah, Ming-Mo Hou, Pedro Ramirez, Lois Ramondetta, Karen Lu, Funda Meric-Bernstam

**Affiliations:** Department of Investigational Cancer Therapeutics, Unit 0455, The University of Texas MD Anderson Cancer Center, 1515 Holcombe Boulevard, Houston, TX 77030 USA; Department of Gynecologic Oncology, The University of Texas MD Anderson Cancer Center, Houston, TX USA

**Keywords:** Vaginal cancer, Vulvar cancer, Phase I trial, Body mass index, Molecular analysis

## Abstract

**Background:**

Early-stage vaginal and vulvar cancer can be cured. But outcomes of patients with metastatic disease are poor. Thus, new therapeutic strategies are urgently required.

**Methods:**

In this retrospective study, we analyzed the clinical outcomes of consecutive patients with metastatic vaginal or vulvar cancer who were referred to a phase I trial clinic between January 2006 and December 2013. Demographic and clinical data were obtained from patients’ electronic medical records.

**Results:**

Patients with metastatic vaginal (*n* = 16) and vulvar (*n* = 20) cancer who were referred for phase I trial therapy had median overall survival durations of 6.2 and 4.6 months, respectively. Among those who underwent therapy (*n* = 27), one experienced a partial response and three experienced stable disease for at least 6 months. Patients with a body mass index ≥30 had a significantly longer median overall survival duration than did those with a body mass index <30 (13.2 months versus 4.4 months, *p* = 0.04). Preliminary data revealed differences in molecular profiling between patients with advanced vaginal cancer and those with advanced vaginal cancer.

**Conclusions:**

Metastatic vaginal and vulvar cancers remain to be difficult-to-treat diseases with poor clinical outcomes. The currently available phase I trial agents provided little meaningful clinical benefits. Understanding these tumors’ molecular mechanisms may allow us to develop more effective therapeutic strategies than are currently available regimens.

## Background

Vaginal and vulvar cancers comprise approximately 8 % of all malignant neoplasms of the female genital tract: approximately 3,000 vaginal cancers and 4,500 vulvar cancers are diagnosed annually in the United States [1]. Most of these tumors are squamous cell carcinomas, but melanoma, sarcoma, adenocarcinoma, and other histological types also occur [2–6]. All vaginal and vulvar cancers are associated with similar risk factors: cigarette smoking, human papillomavirus infection, and a history of other gynecological malignancies [7, 8]. Early-stage vaginal and vulvar cancer can be cured, but if the disease is not amenable to radical local excision or curative chemoradiation therapy [9–11], patients with recurrent or metastatic vaginal or vulvar cancers have a poor prognosis [12, 13]. Palliative systemic therapy results in limited clinical benefit [14].

The overall poor prognosis of these patients warrants the development of novel therapeutic regimens [15]. Therefore, we conducted a retrospective chart review to identify the demographic characteristics and major clinical outcomes, such as mutational status, clinical response, and survival duration, of patients with metastatic vaginal or vulvar cancer who were referred to a designated phase I trial clinic. These data may lead to the development of new drugs for the treatment of patients with these diseases.

## Methods

### Patient selection

We included all consecutive patients with metastatic or recurrent vaginal or vulvar carcinoma who were referred to the Department of Investigational Cancer Therapeutics (a phase I clinical trials program) at The University of Texas MD Anderson Cancer Center between January 1, 2006, and December 31, 2013 in this retrospective chart review. Follow-up was defined as the time of the initial phase I clinic visit until death or the last date of the study, censored on August 8, 2014. This study was conducted in accordance with MD Anderson’s institutional review board guidelines.

### Data collection

During the data collection phase, two members of the research team worked independently: one reviewed patients’ electronic medical records, and the other audited and checked the accuracy of the collected data. Any data discrepancy was resolved by a consensus after group discussion. The collected clinical information included race, treatment history (e.g., surgery, radiation therapy, and chemotherapy), date of birth, Eastern Cooperative Oncology Group performance status at the initial phase I clinic visit, mutation profile of the tumor specimen, phase I clinical trial therapies, and clinical outcomes (progression-free survival [PFS], overall survival [OS], and objective responses, including complete remission [CR], partial response [PR], and stable disease for 6 months or longer [SD ≥6 months]).

Clinical objective responses were evaluated using Response Evaluation Criteria in Solid Tumors software version 1.0 or 1.1, per individual study protocols [16, 17]. The PFS duration was defined as the interval from the date of initial treatment to the first objective documentation of disease progression, the time of death, or the last date of contact (August 8, 2014), at which time the patients’ data were censored. OS duration was estimated from the date of the initial phase I clinical trial therapy to death or the last date of contact. The enrollment of eligible patients into specific phase I trials was dependent on trial availability at the time of presentation and the preference of the treating physician, according to good clinical practice. If a phase I agent was unsuccessful, another was used as long as the patient was eligible and willing to participate.

### Statistical analyses

Categorical data were described using contingency tables. Continuously scaled measures were summarized with descriptive statistical measures (i.e., the median and the range), whereas PFS and OS rates were estimated using the Kaplan-Meier method. Patients who were still alive at the time of data analysis were censored at that time. Fisher’s exact test was used to assess the association between categorical variables. Statistical inferences were based on two-sided tests at a significance level of *p* <0.05. Statistical analyses were carried out using SPSS Statistics software version 22 (IBM, Inc., Armonk, NY).

## Results

### Study population

This study included 36 consecutive patients with metastatic vaginal (*n* = 16) or vulvar (*n =* 20) cancer who were evaluated in the phase I clinic at MD Anderson. The majority of these patients were white (*n =* 32 [89 %]); presented with squamous cell carcinoma (*n =* 24 [67 %]); had adequate functional status, with an Eastern Cooperative Oncology Group performance status of 0 or 1 (*n =* 31 [86 %]); and had undergone systemic chemotherapy (*n =* 30 [83 %]) or radiation therapy (*n =* 32 [89 %]). The patients’ baseline characteristics are listed in Table 1. All patients had undergone systemic chemotherapy or chemoradiation therapy for locally advanced or metastatic disease before they were referred. Most patients (*n =* 27 [75 %]) were enrolled in a phase I trial.

**Table 1:** Baseline patient demographics (*n =* 36)

Characteristics	Vaginal cancer (*n =* 16)	Vulvar cancer (*n =* 20)
Median age, years (range)	60 (30 to 85)	55 (33 to 78)
Race, n (%)		
White	13 (81 %)	19 (95 %)
Black	1 (6 %)	0
Hispanic	2 (13 %)	0
Asian	0	1 (5 %)
Body Mass Index, n (%)		
Underweight (<18.5)	0	1 (5 %)
Normal Weight (18.5 to 25)	10 (62 %)	13 (65 %)
Overweight (>25)	6 (38 %)	6 (30 %)
The Eastern Cooperative Oncology Group Performance Status, n (%)		
0	4 (25 %)	0
1	9 (56 %)	18 (90 %)
2	3 (19 %)	2 (10 %)
Prior Chemotherapy		
Yes, n (%)	14 (88 %)	16 (80 %)
Median number (range)	2 (0 to 4)	1 (0 to 6)
Prior Radiation Therapy		
Yes, n (%)	15 (94 %)	17 (85 %)
Pathological Diagnosis, n (%)		
Squamous Cell Carcinoma	7 (44 %)	17 (85 %)
Adenocarcinoma	4 (25 %)	0
Melanoma	3 (19 %)	3 (15 %)
Carcinosarcoma	1 (6 %)	0
High-grade neuroendocrine carcinoma	1 (6 %)	0
Phase I Trial Enrollment, n (%)	11 (69 %)	16 (80 %)

### Major clinical outcomes

**Fig. 1:**
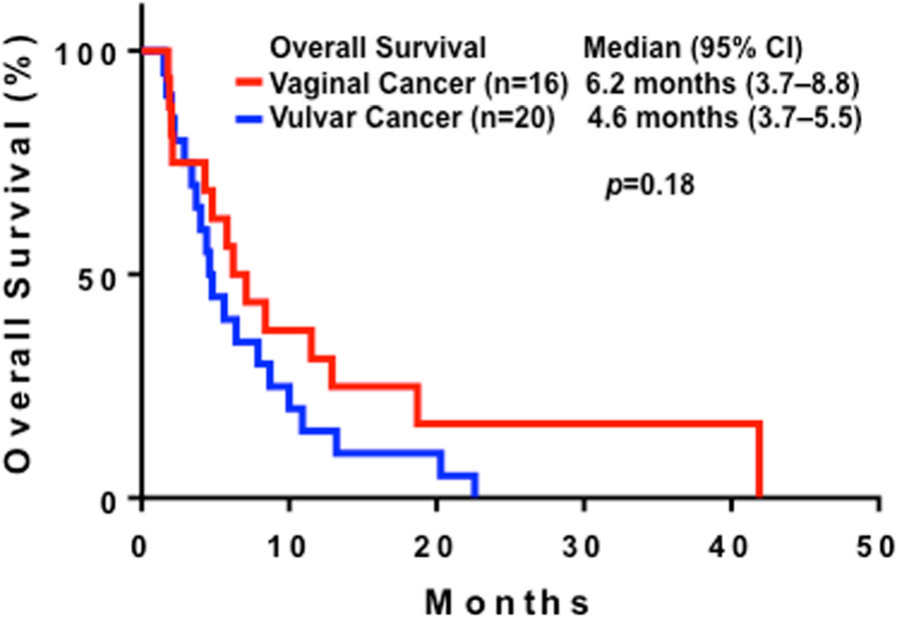
Kaplan-Meier plots of survival in patients with metastatic vaginal cancer (*n* = 16; median, 6.2 months; 95 % CI, 3.7–8.8 months) and vulvar cancer (*n*–20; 4.6 months; 3.7–5.5 months)

The 36 patients had a median OS duration of 5.6 months (95 % CI, 3.7–7.5 months). A similar duration was observed in patients with vaginal cancer (6.2 months; 3.7–8.8 months) and vulvar cancer (4.6 months; 3.7–5.5 months (*p* = 0.18), as shown in Fig. 1.

**Fig. 2:**
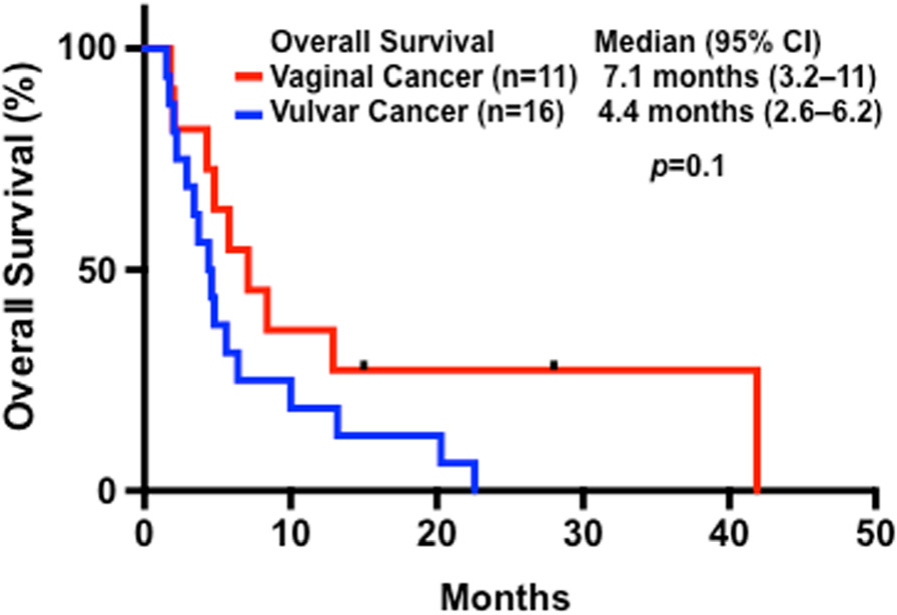
Kaplan-Meier plots of survival in patients with metastatic vaginal cancer (*n* = 11; median, 7.1 months; 95 % CI, 3.2–11 months) and vulvar cancer (*n* = 16; 4.4 months; 2.6–6.2 months) who underwent phase I trial therapy

**Fig. 3:**
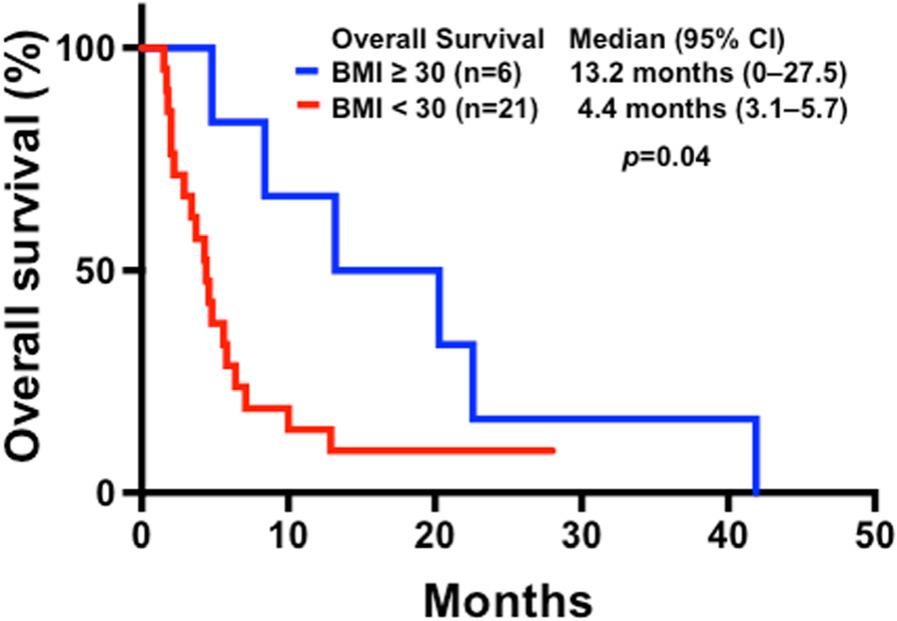
Kaplan-Meier plots of survival in patients with metastatic vaginal or vulvar cancer and a body mass index (BMI) of ≥30 (*n* = 6; median, 13.2 months; 95 % CI, 0–27.5 months). These patients survived significantly longer than did those whose BMI was <30 (*n* = 21; 4.4 months; 3.1–5.7 months) (*p* = 0.04)

The baseline demographics, major clinical outcomes, and molecular aberrations of the 27 patients who underwent phase I trial therapy, are listed in Table 2, associated with a median OS duration of 5.6 months (95 % CI, 3.1–8.1 months). There was no difference in the median OS duration between patients with vaginal cancer (7.1 months; 3.2–11 months) and vulvar cancer (4.4 months; 2.6–6.2 months; *p* = 0.1), as shown in Fig. 2. In this cohort of patients who underwent phase I trial therapy, six (22 %) were classified as obese (BMI ≥30), one (4 %) overweight (BMI 25–30), 19 (70 %) normal (BMI 18.5–25), and one (4 %) underweight (BMI <18.5). Obese patients had a median OS duration of 13.2 months (95 % CI, 0–27.5 months), which was significantly longer than that of those who were not obese (4.4 months; 3.1–5.7; *p* = 0.04), as shown in Fig. 3.

**Table 2:** Major characteristics and clinical outcomes in patients receiving a phase I trial therapy (*n =* 27)

Age	Pathology	Prior therapy	OS	BMI	MDACC score	Phase I trials	PFS	CMS46 or Foundation Med
Vaginal cancer								
45	A	2	41.9	33.2	1	Bevacizumab and Temsirolimus	1.0	ND
81	S	0	5.8	21.9	0	Gemcitabine and Dasatinib	0.9	ND
61	M	4	2.0	23.0	3	PI3K Inhibitor and Paclitaxel	0.9	ND
59	A	3	4.8	37.8	2	Bevacizumab and Temsirolimus plus Carboplatin	PFS1 = 1.4	ND
						CHK1 Inhibitor	PFS2 = 1.5	
						Erlotinib and Pralatrexate	PFS3 = 2.2	
53	S	2	12.9	18.6	0	Aurora Kinase Inhibitor	4.9	ND
57	S	1	1.8	22.9	2	Trientine and Carboplatin	0.7	PIK3CA (E545K)
57	S	0	28+	21.3	1	Everolimus and Pazopanib	PFS1 = 18.2	PIK3CA (E545K), PTPRD (S1845fs*2) and STK11 loss
						PI3K Inhibitor	PFS2 = 1.9	
72	M	1	4.3	21.0	1	Ipilimumab and Imatinib	2.3	PTEN loss, C17orf39, KDR, KIT and MYST3 amplification
58	S	1	15+	19.5	1	Erlotinib and Pralatrexate	14.6+	ERBB2 (S310F), ERBB4 (D609N), FBXW7 (R479Q), RB1 (E539*), ARID2 (Q1194*) and amplification of EPHBI, PIK3CA and SOX2
67	A	2	8.4	31.3	1	Anastrozole and Everolimus	2.6	PTEN (210-1G > A), KRAS (G12V), CTNNB1 (D32N), MPL (P106L), and amplification of MCL1, MYC and NFKB1A
52	S	1	7.1	24.0	0	Erlotinib and Valproic Acid	2.8	ND
Vulvar cancer								
37	S	1	3.7	21.2	2	PI3K inhibitor plus Caboplatin and Paclitaxel	PFS1 = 1.4	PIK3CA: mutation not detected
						Erlotinib and Valporic acid	PFS2 = 0.6	
58	S	1	13.2	30.5	1	Erlotinib and Valporic acid	PFS1 = 3.9	BRAF, KRAS and PIK3CA: no mutation detected
						Bevacizumab and Cetuximab plus Erlotinib	PFS2 = 7.2	
74	S	1	6.4	23.7	1	Microtube Inhibitor	3.1	ND
60	M	6	4.4	22.3	2	PI3K Inhibitor	2.0	Single Gene: c-KIT (L576P)
42	S	1	2.0	23.7	1	Bevacizumab and Trastuzumab plus Lapatinib	0.7	ND
42	S	0	1.5	19.9	2	Src Inhibitor	0.5	ND
78	S	0	20.3	35.5	1	Erlotinib and Valporic Acid	6.1	ND
37	S	1	1.7	18.7	2	Camptothecin	0.8	ND
55	S	3	4.8	15.8	1	Histone Deacetylase Inhibitor	0.5	ND
41	S	0	2.2	18.8	2	Sirolimus and Docetaxel	1.7	ND
54	S	1	2.9	26.6	1	Lapatinib and Sirolimus	1.5	Single Gene: BRAF (V600E)
60	M	2	4.6	22.9	3	Multikinase Inhibitor	2.9	ND
69	S	1	22.6	30.9	2	Carboplatin and Trientine	0.9	A 46-gene panel: no mutation detected
33	S	1	10.0	24.8	2	Lenalidomide and Temsirolimus	1.9	ND
73	S	1	5.6	22.4	2	Crizotinib and Pazopanib	1.6	KRAS (R102T), TET2 (W1198*), TP53 (R248Q), and CDK2NA/B loss
55	M	1	3.4	24.2	2	Translation Initiation Inhibition	1.0	ND

*OS* overall survival, *BMI* body mass index, *MDACC score* the sum of five variables (low serum albumin, high serum lactate dehydrogenase, *ECOG performance status of 1 or higher* more than two metastatic sites, and gastrointestinal tumor type), *PFS* progression-free survival (1, 2, or 3 indicates the first, second, or third line of phase I trial), * deletion , *A* adenocarcinoma, *S* squamous cell carcinoma, *M* melanoma, *ND* not done

### Exploratory molecular analysis

Molecular marker studies were performed of available tumor specimens in a Clinical Laboratory Improvement Amendments-certified molecular diagnostic laboratory. Table 3 lists the limited molecular aberrations per tumor type. In patients with metastatic vaginal cancer, the PI3K/AKT/mTOR pathway was activated, which was supported by the finding of frequent *PIK3CA* mutations and *PTEN* loss or mutation. A loss of *STK11* and *FBXW7* mutations was also observed in these patients. Furthermore, treatment with everolimus and pazopanib led to a partial response for 18.2 months in one patient with metastatic squamous cell carcinoma that harbored PIK3CA (E545K), PTPRD (S1845fs*2), and STK11 losses.

**Table 3:** Summary of molecular aberrations per tumor type

	Vaginal cancer, *n* (%)	Vulvar cancer, *n* (%)
PIK3CA	33 % (*n =* 9)	0 % (*n* = 8)
PTEN	67 % (*n* = 6)	0 % (*n* = 4)
KRAS	10 % (*n* = 10)	13 % (*n* = 8)
NRAS	10 % (*n* = 10)	0 % (*n* = 4)
EGFR	0 % (*n* = 9)	0 % (*n* = 6)
BRAF	0 % (*n* = 9)	14 % (*n* = 7)
C-MET	0 % (*n* = 7)	0 % (*n* = 3)
TP53	0 % (*n* = 6)	33 % (*n* = 3)
C-KIT	0 % (*n* = 8)	20 % (*n* = 3)

Treatment with erlotinib and pralatrexate resulted in stable disease for approximately 15 months in a patient with metastatic squamous cell carcinoma that harbored ERBB2 (S310F), ERBB4 (D609N), FBXW7 (R479Q), RB1 (E539*), and ARID2 (Q1194*) and amplification of EPHBI, PIK3CA, and SOX2. In patients with metastatic vulvar cancer, we found no common intracellular transduction pathway mutations; rather, mutations were found in *TP53*, *c-KIT*, *BRAF*, and *KRAS*. One patient with metastatic KRAS wild-type squamous cell carcinoma experienced stable disease for about 11 months after being treated with epithelial growth factor inhibition-based phase I trial regimens, while another patient had stable disease for 6 months after being treated with erlotinib and valproic acid.

## Discussion

Patients with metastatic or recurrent vaginal or vulvar cancer have limited therapeutic treatment options [18–20]. In this study, we found that these patients did not experience a meaningful clinical benefit from novel phase I therapeutics: there were low rates of objective responses and a median OS duration of only 5.6 months. Further evaluation is warranted to determine the effects of novel cancer therapeutics, molecular profiling, and targeted therapy on patient outcomes in the phase I setting.

There were several notable observations in our study. In general, patients with metastatic vaginal squamous cell carcinoma had a median OS duration of 7.1 months compared with 4.4 months in those with metastatic vulvar squamous cell carcinoma. Both cohorts of patients had poor clinical outcomes and low antitumor activity in response to currently available phase I agents. These patients had significantly shorter OS durations than did other patients with other metastatic or recurrent solid tumors [21–23]. Second, patients with metastatic vaginal cancer had a higher prevalence of PI3K/AKT/mTOR pathway activation, while patients with metastatic vulvar cancer had no common transduction pathway mutations. Therefore, early molecular profiling is urgently required to further explore therapeutic options for these patients. Third, since the association between obesity and survival in patients with metastatic gynecological malignancies remains equivocal, we determined the relationship between BMI and major clinical outcomes. Patients with BMIs of ≥30 had a significantly longer median OS duration (13.2 months) than did those with BMIs <30 (4.4 months), suggesting that further studies are warranted of the effects of excess body weight on tumor biology. Elucidating the molecular mechanisms of vaginal and vulvar cancer may result in the development of more effective therapeutic strategies [24, 25].

A limited sample size was available for subgroup analyses, which confounded our ability to validate statistical significance in the category assessment. Nevertheless, the findings of this retrospective study should be considered preliminary evidence for generating hypotheses that will require further validation in larger prospective studies.

## Conclusion

In conclusion, metastatic vaginal and vulvar cancers are difficult-to-treat diseases with poor clinical outcomes. The currently available phase I trial agents provided little meaningful clinical benefits. Preliminary data revealed differences in molecular profiling between patients with advanced vaginal cancer and those with advanced vaginal cancer. Therefore, we advocate the earlier use of molecular profiling to obtain a better understanding of their tumorigenesis and development. Biomarker-driven therapies based on complex molecular profiles may be an initial step to develop effective therapeutic regimens treating these malignancies.
